# Photon-deficient Mass on FDG-PET Scan in Renal Cell Carcinoma: A Case Report

**DOI:** 10.4137/ccrep.s724

**Published:** 2008-12-23

**Authors:** George Shih, Wei-Jen Shih, Bonnie Mitchell, Primo P Milan

**Affiliations:** Department of Radiology, Weill Medical College, Cornell University New York, NY, and Nuclear Medicine Service, Pathology Service and Radiology Service, Lexington VA Medical Center, and Department of Diagnostic Radiology and Department of Pathology, College of Medicine, University of Kentucky, Lexington, Kentucky, U.S.A.

**Keywords:** renal cell carcinoma, photon-deficiency, F-18 FDG-PET, MR I, CT, U.S., tumor necrosis, glycolysis

## Abstract

F-18 Fluorodeoxyglucose Positron Emission Tomography imaging (F-18 FDG PET) detects malignancies depending on the uptake profile of glycolysis of tumors; however, the role of FDG PET is limited in the evaluation of primary renal malignancy because of low FDG uptake by renal cell carcinoma and also because normal urinary excretion of FDG seen in the images. A patient with renal cell carcinoma whose FDG PET imaging study incidentally shows a photon-deficient mass in the upper pole of the right kidney is present here. The diagnosis is also validated by the histopathological findings of tumor necrosis, hemorrhage, and scars.

## Introduction

It is well established that advances in F-18 FDG PET imaging may lead to early cancer detection, more accurate tumor staging and consequently adequate treatment, better monitoring of the disease and enhanced surveillance for recurrences after treatment. The role of FDG PET is limited in the evaluation of primary renal malignancy because of low FDG uptake by renal cell carcinoma (RCC) and because of the normal urinary excretion of FDG seen in the images.^1^,^2^ Here, a photon-deficiency lesion in the right kidney found on FDG PET is reported.

## Case Report

**Figure 1:**
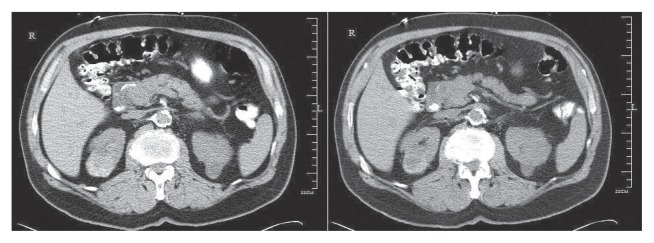
CT images of the abdomen with contrast medium shows a heterogeneously enhancing exophytic mass projecting posteriorly from the upper pole of the right kidney.

**Figure 2:**
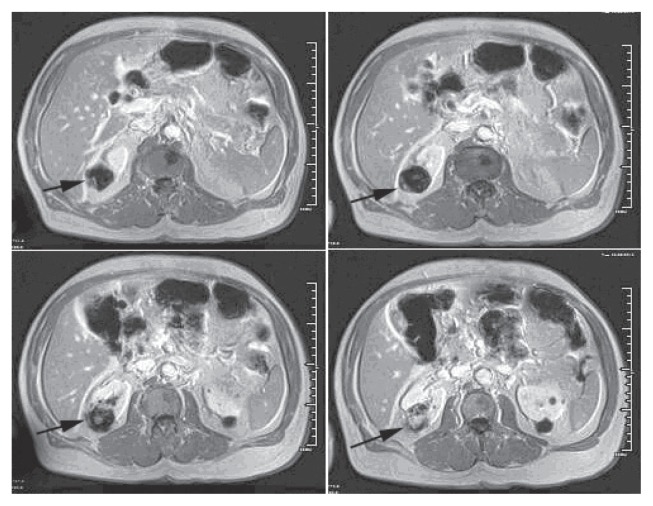
MR images with contrast showed a 4-cm complex enhancing mass at the projecting from the posterolateral aspect of the upper pole (indicating arrows) of the right kidney; the complex mass demonstrated heterogeneous signal internally including a focal hypointense signal on precontrastT1 weighted sequences; and these areas also showed hyperintense signal postcontrast suggesting hemorrhage, necrosis, and scar formation components.

**Figure 3:**
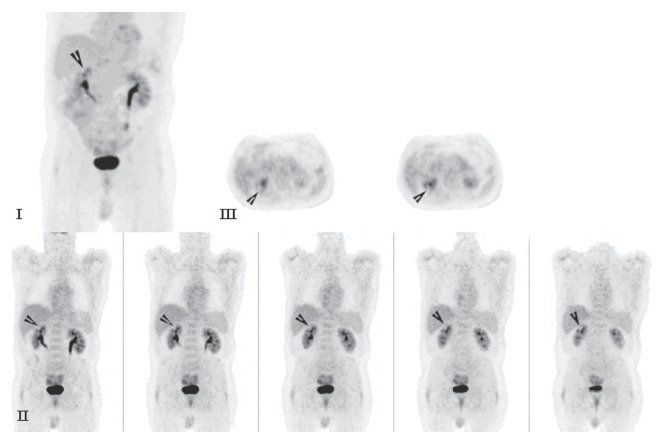
F-18 FDG PET images. I. Maximal intensity image shows a photon-deficient area in the upper pole of the right kidney as indicated by an open-arrowhead. II. Coronary slices show a photon-deficient area in the upper pole posterior-laterally as indicated by open-arrowheads. III. Transverse slices show a photon-deficient area in the upper pole posterior-laterally as indicated by opnen-arrowheads.

A 69-year-old man with a history of having undergone coronary artery bypass graft, cholecystectomy, cerebro-vascular accident with craniotomy and recent hoarseness of voice for eight months had recently invasive squamous cell carcinoma of vocal cord. CT of the neck without contract enhancement showed deformity of the larynx with thickening of the anterior commissure and right vocal cord which are characteristic of a mucosal lesion. He also suffered from new onset of changes in non-specific gastrointestinal symptoms. Therefore he underwent an abdominal CT with contrast medium which incidentally showed a heterogeneously enhancing exophytic mass projecting posteriorly from the upper pole of the right kidney (Fig. 1). Four days later, ultrasonic (U.S.) of kidney showed a solid mass measuring 4-cm in the upper pole of the right kidney. Three weeks later, an MRI with contrast medium showed a 4-cm complex enhancing mass projecting from the posterolateral aspect of the upper pole of the right kidney; the complex mass demonstrated heterogeneous signal internally including a focal hyperintense signal on precontrastT1 weighted sequences; and these areas also showed hyperintense signal postcontrast suggesting hemorrhage, necroses, and scar formation (Fig. 2). F-18 FDG PET images showed two small areas of faintly increased activity in the larynx (not shown); in addition, a photon-deficient area was also seen in the upper border of the right kidney (Fig. 3). Subsequently, a right nephrectomy using hand-assisted laparoscopic nephrectomy directed laparocopy with biopsy and direct laryngoscopy with biopsy were same day resulted in clear renal cell carcinoma with focal papillary features (Figs. 4A and B) an focal capsular invasion approxmimal one-two cell layers short of true transcapular involvement us present. In addition, areas of hemorrhage, tumor necrosis, and scars (4C and D) were noted.

## Discussion

A case of a photon-deficient area in the upper pole of the right kidney on FDG PET scan (Fig. 2), which was subsequently confirmed by CT and MRI imaged (Figs. 1 and 2), and diagnosed as RCC as presented here. This finding of “cold” or photon-deficient area in the right kidney seen in FDG PET images (Fig. 3) has not been previously reported. The photon-deficient area in the right kidney of this patient might be partially explained by tumor necrosis, hemorrhage and fibrosis/scar tissue (Fig. 4) which were shown on MR images as well.

RCC is a diagnostic challenge. Among all patients with RCC, 25%–39% are asymptomatic and the diagnosis is made from a radiological study obtained for the reasons.^1^,^2^ This patient showed no clinical symptom related to RCC which, was detected serendipitously by abdominal CT imaging (Fig. 1) as part of working up for a new onset of non-specific gastro-intestinal symptoms. Incidentally, CT imaging of the abdomen with contrast medium (Fig. 1) showed the lesion in the upper pole of the right upper. An ultrasonogram confirmed a solid mass in the right upper pole of the kidney; subsequently MRI showed a 4-cm complex mass at the superior-posterior aspect of the right kidney (Fig. 2).

In the study of FDG PET in detection of RCC, Ak and Can reported that F-18 FDG PET may have a role in the diagnosis and evaluation of patients with RCC and primary staging of the disease.^3^ However, usually FDG is excreted by kidneys and it is difficult to distinguish FDG excreted in the urine from that accumulating in the tumor unless the patient has renal failure with no urine formation. Visualization is difficult in a patient with RCC who has renal failure and is on long-term hemodialysis because FDG is not excreted in the urine.^4^ Furthermore, the detection rate of RCC by PET is generally as low as 31.5%–60%.^5^,^6^ The role of FDG PET in the detection of RCC is limited by low sensitivity^7^,^8^ because isometabolism of renal tumors has been reported^9^ and FDG PET displays poor efficacy in cases of renal cell carcinoma because of low levels or absence of glycolysis in this type of tumor.^10^ An unusual case of photon-deficiency in renal tumor (RCA) is reported here.

FDG PET is limited in the evaluation of primary urological malignancies including prostate and urinary bladder cancer.^11^,^12^ It has been reported that FDG PET does not show primary renal tumors, and is more useful for detecting postoperative local recurrence or distal metastasis than the primary tumor.^13^ In the evaluation of distant metastases from RCC, FDG PET in not a sensitive imaging modality and may not adequately characterize small metastatic lesion.^14^ FDG study might demonstrate tumor invasion of the right renal vein and infra-hepatic inferior vena cava.^15^–^17^

In summary, a patient with renal cell carcinoma, whose FDG PET incidentally showed a photon-deficient mass in the upper pole of the right kidney is presented; this finding can be explained by non- uptake of the tracer by the tumor, and the histopathological findings of tumor necrosis, hemorrhage, and scars.

## Figures and Tables

**Figure 4A:**
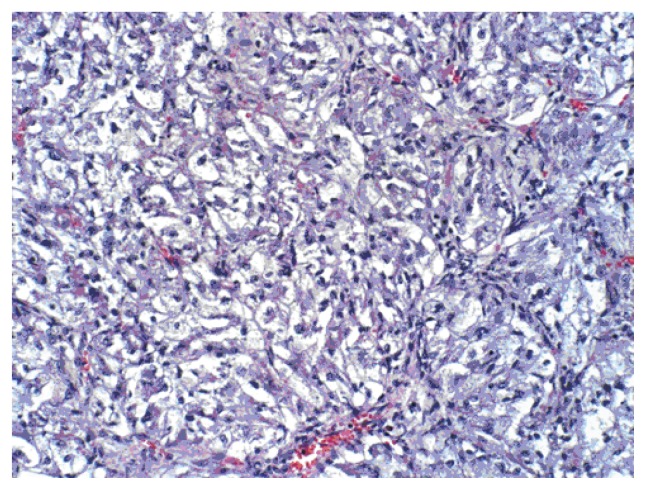
Tumor is composed of clear cells.

**Figure 4B:**
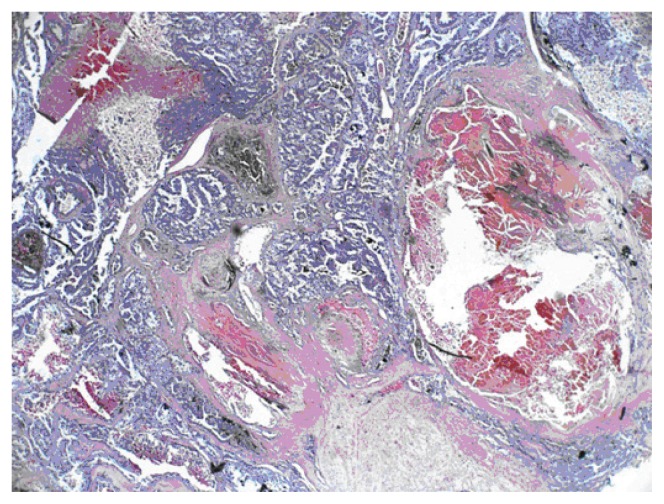
Focal papillary features with areas of hemorrhages, tumor necrosis, and scar formations.

**Figure 4C:**
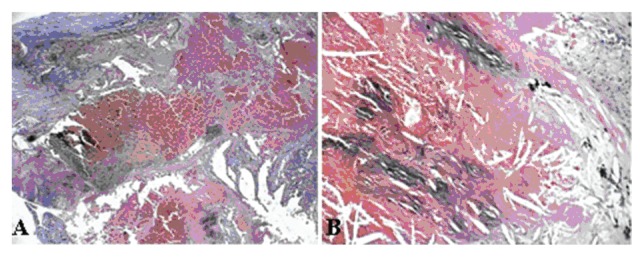
Areas of hemorrhages and scar formation.

**Figure 4D:**
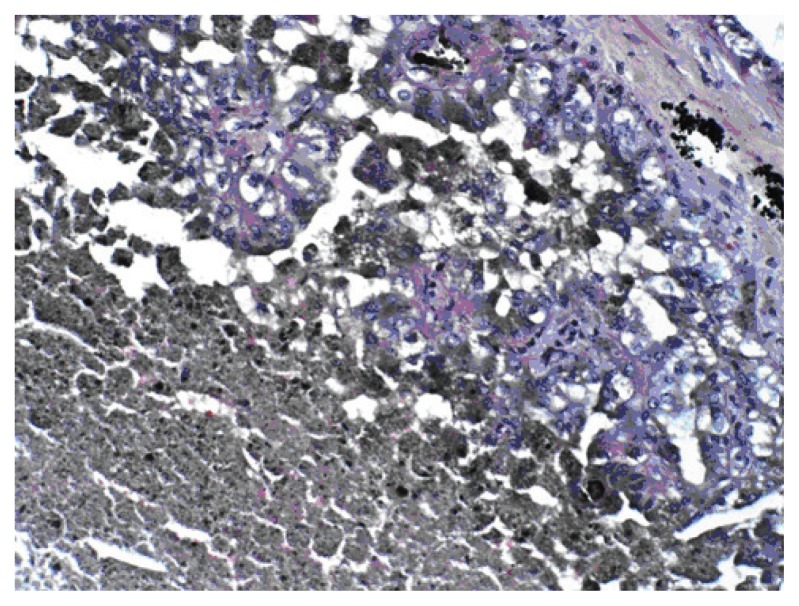
Tumor necrosis.
